# Follicular-Phase GnRH Agonist Protocol Is Another Choice for Polycystic Ovary Syndrome Patients With Lower LH/FSH and Lower AMH Levels Without Increasing Severe OHSS Risk

**DOI:** 10.3389/fendo.2022.905263

**Published:** 2022-06-10

**Authors:** Rui Gao, Xin Liao, Wanrong Huang, Rujun Zeng, Lang Qin, Peng Bai

**Affiliations:** ^1^The Reproductive Medical Center, Department of Obstetrics and Gynecology, West China Second University Hospital, Sichuan University, Chengdu, China; ^2^Key Laboratory of Birth Defects and Related Diseases of Women and Children of the Ministry of Education, West China Second University Hospital, Sichuan University, Chengdu, China; ^3^West China School of Medicine, Sichuan University, Chengdu, China; ^4^Department of the Central Operating Unit, West China Second University Hospital, Sichuan University/West China School of Nursing, Sichuan University, Chengdu, China; ^5^Department of Dermatology, The First Hospital of China Medical University, Shenyang, China; ^6^Department of Forensic Genetics, West China School of Basic Medical Sciences & Forensic Medicine, Sichuan University, Chengdu, China

**Keywords:** controlled ovarian stimulation (COS), polycystic ovarian syndrome, *in vitro* fertilization, intracytoplasmic sperm injection (ICSI), ovarian hyperstimulation syndrome (OHSS), clinical pregnancy rate (CPR)

## Abstract

**Purpose:**

To explore another choice for a controlled ovarian stimulation (COS) protocol that does not increase severe ovarian hyperstimulation syndrome (OHSS) risk among polycystic ovarian syndrome (PCOS) patients with specific clinical features.

**Methods:**

A retrospective study was performed. Two hundred and fifty-nine participants were divided into two groups, group 1 (fixed GnRH antagonist protocol, n = 295) and group 2 (follicular-phase GnRH agonist protocol, n = 69) according to COS protocols. The basic characteristics and laboratory indicators between these two groups were compared. The severe OHSS rate and clinical pregnancy rate were selected as indicators to evaluate the risks and benefits of the two COS protocols. Subgroup analyses for the severe OHSS rate and clinical pregnancy rate were performed based on baseline luteinizing hormone/follicle-stimulating hormone (bLH/FSH) and anti-Mullerian hormone (AMH) levels.

**Results:**

The severe OHSS rate was statistically higher in group 2 than in group 1 (11.6% vs. 3.7%, p = 0.008), but the biochemical pregnancy rate and clinical pregnancy rate showed no statistical difference between the groups (71.9% vs. 60.3% and 62.5% vs. 54.3%). In the higher bLH/FSH subgroup (≥1.33) and the higher serum AMH level subgroup (>3.4 ng/ml), severe OHSS incidence was statistically higher in group 2 compared to group 1, but this incidence was lower in the bLH/FSH subgroup (<1.33) and the subgroup with lower serum AMH levels (≤3.4 ng/ml); a difference in severe OHSS risk was not observed. There was no statistical difference between the two groups regarding clinical pregnancy rate in any subgroup.

**Conclusion:**

The limited evidence from this study indicates that in PCOS patients with lower bLH/FSH levels (<1.33) and lower serum AMH levels (≤3.4 ng/ml), a follicular-phase GnRH agonist protocol may be another choice that does not increase the risk of severe OHSS.

## Introduction

Polycystic ovary syndrome (PCOS) is one of the most common and heterogeneous endocrinological problems among women of reproductive age (1). PCOS affects more than 10% of women around the world (1), and the prevalence of PCOS in Chinese women aged 19–45 years old is 5.6% (2). The clinical manifestations of PCOS are complicated and individualized. According to the Rotterdam criteria, a diagnosis of PCOS must include at least two of the following three features: oligo-anovulation, clinical and/or biochemical hyperandrogenism, and polycystic ovaries on ultrasonography, excluding other endocrinopathies (3). Thus, irregular menstruation, amenorrhea, hairiness, acne, a higher baseline antral follicle count (AFC; the presence of 12 or more follicles in each ovary measuring 2–9 mm in diameter), or increased ovarian volume (>10 ml) are always observed in PCOS patients. In addition, other clinical manifestations include a higher baseline luteinizing hormone/follicle-stimulating hormone ratio (bLH/FSH) (4, 5), higher levels of anti–Müllerian hormone (AMH) (5, 6), insulin resistance (7), and obesity, all of which are common among PCOS patients despite not being included in the diagnosis criteria for PCOS.

About 80% of anovulation infertility is caused by PCOS according to the European Society of Human Reproduction and Embryology (ESHRE) and the American Society for Reproductive Medicine (ASRM) (3). For PCOS patients suffering infertility, assisted reproductive technology (ART) is an important strategy for achieving pregnancy (8). Controlled ovarian stimulation (COS) is an essential step for *in vitro* fertilization (IVF) and/or intracytoplasmic sperm injection (ICSI), as its purpose is to induce the maturation of more oocytes, thus maximizing the chance of successful pregnancy. Ovarian hyperstimulation syndrome (OHSS) is an uncommon but serious complication associated with COS, and evidence from well-designed cohort or case–control studies indicates that PCOS is a risk factor of OHSS; it may also be related to higher ovarian reserve markers such as elevated AMH levels, peak estradiol levels, and higher AFC (9). Previous studies have demonstrated that a gonadotropin-releasing hormone (GnRH) antagonist protocol can reduce the risk of OHSS (10–12). Thus, the GnRH antagonist protocol is recommended as a first-line COS protocol for PCOS patients, according to the World Health Organization (WHO) (13). No high-quality, randomized, controlled trials providing direct evidence for COS selection in PCOS patients have, however, been performed, and some studies also suggest that the GnRH antagonist protocol might result in lower cumulative live birth rates (cLBRs) (14) and lower ongoing pregnancy rates (12) compared to a GnRH agonist protocol used in fresh embryo transfer cycles. Failure in IVF/ICSI is a psychological stressor for women, and patients with poor IVF/ICSI outcomes are more likely to suffer anxiety and depression (15, 16). Improving clinical pregnancy rates, ongoing pregnancy rates, and LBR without increasing the incidence of OHSS is a key point of IVF/ICSI, as this balances the risk of OHSS against clinical benefit.

Some demographic characteristics and biomarkers were found to have predictive value for severe OHSS, and among them, convenient peripheral blood biomarkers associated with ovarian reserves such as AMH and bLH/FSH have been widely used to predict the risk of OHSS. For example, a previous study showed that an AMH value over 3.4 ng/ml is an independent risk factor for severe OHSS, but for PCOS patients with AMH values less than or equal to 3.4 ng/ml, severe OHSS is acceptable (9). Thus, whether the GnRH antagonist protocol leads to lower severe OHSS rates compared to the GnRH agonist protocol in PCOS patients with lower AMH and lower bLH/FSH levels is a point of confusion that has not been investigated by previous studies. In addition, the possibility of clinical pregnancy is worth considering, especially for patients with a history of recurrent IVF/ICSI failure. Clinical pregnancy outcomes directly depend on COS protocols in IVF/ICSI and fresh embryo transfer cycles, but for PCOS patients, the available COS protocols are restricted because of severe OHSS risks. The effect of follicular-phase GnRH agonist protocols on clinical pregnancy outcomes in PCOS patients with lower AMH and lower bLH/FSH levels have not been explored. Therefore, in this retrospective study, the severe OHSS rate and clinical pregnancy rate of PCOS patients receiving a fixed GnRH antagonist protocol and a follicular-phase GnRH agonist protocol—based on subgroups classified by AMH and bLH/FSH levels—were evaluated, thereby providing more evidence for the selection of individualized COS protocols for PCOS patients.

## Materials and Methods

### Participants and Study Design

A retrospective cohort study was performed by analyzing the records of PCOS patients who had entered their first cycle undergoing standard IVF/ICSI due to infertility at the Reproductive Center of West China Second University Hospital, Sichuan University, Chengdu, China, from June 2020 to June 2021. Infertility is defined as a disease of the reproductive system characterized by the failure to achieve a clinical pregnancy after 12 months or more of regular, unprotected sexual intercourse. Only patients who underwent a fixed GnRH antagonist protocol and follicular GnRH agonist protocol were included. Patients were divided into two groups according to their COS protocols. PCOS among the patients was diagnosed according to the Rotterdam criteria. Patients with a history of genital tuberculosis, a history of recurrent pregnancy loss, a history of ovarian surgery, evidence for hyper-prolactinoma or hypothyroidism, and other associated infertility factors, except for tubal factors, were excluded. Patients receiving preimplantation genetic diagnoses were also excluded. Ultimately, 295 patients received the fixed GnRH antagonist protocol (group 1), and 69 patients received the follicular phase GnRH agonist protocol (group 2). All these patients met the inclusion criteria and were included in this study. This study was performed according to the World Medical Association Declaration of Helsinki and was approved by the Ethics Committee of West China Second University Hospital.

### Controlled Ovarian Stimulation Protocol

In the fixed GnRH antagonist protocol, patients were started on intramuscular injections of recombinant FSH (injection Gonal-f, Merck Serono Specialties, Italy) from the second day of their menstrual cycle. The starting dose was between 150 and 225 IU/day. A GnRH antagonist (injection Cetrotide acetate, Aeterna Zentaris, Canada) was administered at a dose of 0.25 mg/day from the sixth day of the menstrual cycle until the ovulation trigger day. In the follicular phase GnRH agonist protocol, transvaginal ultrasounds (TVS) were performed on the second or third day of the menstrual cycle to assess AFC, and the intramuscular injection of the GnRH agonist (Triptorelin; Ferring, Kiel, Germany) was commenced at 3.75 mg if no follicle reached 10 mm in diameter. Twenty-eight days later, a serum sex hormone assessment and TVS were performed, and pituitary downregulation was completed if the patients met the following criteria: E2 ≤ 30 pg/ml, LH ≤ 5 IU/l, ovarian follicle diameter ≤ 5 mm, and endometrial thickness (ET) ≤ 5 mm. The intramuscular injection of recombinant FSH was provided (150 to 225 IU/day) for ovarian stimulation based on patient age, BMI, and AFC. The dose of recombinant FSH was adjusted every 3–4 days according to the ovarian response until the trigger day. These cycles were cancelled in patients with no follicle greater than 10 mm in diameter after 10 days of recombinant FSH stimulation. For all these protocols, when at least two follicles reached 18 mm or three follicles reached 17 mm, the final stage of triggering ovulation was performed using human chorionic gonadotropin (hCG; Lizhu Pharmaceutical Trading, Zhuhai, China) at doses from 8,000 to 10,000 IU. For women at a high risk for OHSS, low doses of hCG (4,000 to 5,000 IU) were used to trigger ovulation. Serum sex hormone levels and ET were measured on the trigger day.

### Oocyte Retrieval and Embryo Transfer

Oocyte retrieval was performed 36–38 h after triggering ovulation by transvaginal-guided, single-lumen needle aspiration. Oocyte assessment was performed by the standard morphology criteria (17), and a nuclear maturity assessment was performed for cases subjected to ICSI. Conventional IVF or ICSI was performed depending on semen parameters and previous fertilization history. Ultrasound guidance was used for all embryo transfers and was performed 3 or 5 days after oocyte retrieval. Embryo transfer was cancelled for severe OHSS or high OHSS risk (peak E2 > 4,500 pg/ml) patients, and all embryos were frozen. Severe OHSS was diagnosed *via* clinical evidence of ascites and/or hydrothorax, severe dyspnea, oliguria/anuria, intractable nausea/vomiting, severe hemoconcentration (Hct > 55%), a white cell count over 25 × 10^9^/l, creatinine clearance (CrCl) < 50 ml/min, creatinine (Cr) > 1.6 mg/dl, sodium (Na+) <135 mEq/l, potassium (K+) > 5 mEq/l, and elevated liver enzymes according to the ARSM guidelines for OHSS (9).

All patients were given luteal phase support *via* the intramuscular injection of progesterone at 100 mg/day. Two weeks after embryo transfer, pregnancy was assessed by serum β-hCG assay (where serum β-hCG > 50 IU/l was regarded as biochemical pregnancy) and confirmed *via* TVS after another 2 weeks (the presence of the gestational sac was regarded as clinical pregnancy). The measurement of E2, progesterone, LH, FSH, and βhCG was done by fully automated electro-chemiluminescence technology (Roche Cobas e411 analyzer, Hitachi, Tokyo, Japan).

### Information Collection

Basic patient information on age; height; weight; BMI; the duration of infertility, the type of infertility (primary infertility or secondary infertility); basic serum FSH, LH, E2, and P levels (*via* detection on the second day of the menstrual cycle); serum levels of AMH and T; and AFC were collected from hospital records. PCOM was defined as the presence of 12 or more follicles in each ovary measuring 2–9 mm in diameter and/or increased ovarian volume (>10 ml). Information associated with COS was also collected, including information on the rate of OHSS, the Gn starting dose, the total number of Gn days, the total Gn dose, and serum E2, P, and LH levels on the day of hCG, ET on the day of hCG, the number of follicles greater than or equal to 14 mm in diameter, the number of oocytes retrieved, the number of MII oocytes, the 2PN number, the fertilization rate, the cleavage rate, the number of available D3 embryos, the number of high-quality D3 embryos, the number of available blastocysts, the number of high-quality blastocysts, and the rate of cancelled cycles. The MII oocyte rate was defined as the percentage of MII oocytes among the total number of oocytes retrieved. The cleavage rate was defined as the percentage of cleavage embryos among the total number of zygotes. The high-quality D3 embryo rate was defined as the percentage of high-quality D3 embryos among the total number of normal cleavage embryos. The available D3 embryo rate was defined as the percentage of available D3 embryos among the total number of cleavage embryos. The high-quality blastocyst rate was defined as the percentage of high-quality blastocysts among the total number of cleavage embryos used in blastocyst cultures. Moreover, the available blastocyst rate was defined as the percentage of available blastocysts among the number of cleavage embryos used in blastocyst cultures. Embryo grading was done by standard morphology assessment according to modified Veecks’ scoring (18). Blastocysts graded as AA, AB+, AB−, B+A, B−A, B+B+, and BB were classified as high-quality embryos. Fertilization was defined as the presence of pronuclei 16–18 h after insemination/injection. Primary outcome measures consisted of the severe OHSS rate and the clinical pregnancy rate (defined as the presence of a gestational sac per ET).

### Statistical Analysis

Multiple imputation by chained equations was used for missing values in the covariates of the adjusted statistical models. This was performed in the study population and was conducted separately for each group. Continuous variables were expressed as medians (interquartile range) and were compared *via* the Mann–Whitney U test. Categorical measurements are presented as a percentage, and these rates were compared *via* the chi-squared test; if numbers were less than 5 in at least 20% of the cells, Fisher’s exact test was performed. The study population was stratified by bLH/FSH (<1.33 and ≥1.33) and AMH (≤3.4 and >3.4 ng/ml), both of which were reported as risk factors for severe OHSS in previous studies. In addition, cutoff values were also made according to previous studies or guidelines (4, 9, 19–21), and if events in two or more subgroups were zero, the related indicator was excluded from subgroup analysis. The differences between two groups were presented as odds ratios (OR) and 95% confidence intervals (CI). P values less than 0.05 were considered statistically significant, but P values less than 0.1 were also noted. Statistical analyses were performed by SPSS, version 25.0 (SPSS Inc., Chicago, IL, UPL).

## Results

### Basic Participant Information

Basic information on the participants in this study is shown in **Table 1**. There were no statistical differences in age, height, weight, BMI, the duration of infertility, the type of infertility, T serum levels, serum sex hormone levels on days 2–3 of the menstrual cycle, and the fertilization type between the two groups. The prevalence of irregular menstruation was, however, higher in group 1 than in group 2 (90.2% vs. 81.2%, p = 0.035), and the levels of serum AMH were significantly higher in group 1 than in group 2 [10.42 (6.24–145.23) vs. 6.03 (3.61–10.20), p < 0.001]. Also, the prevalence of PCOM was higher in group 1 than in group 2 (68.1% vs. 46.4, p = 0.001).

**Table 1:** Basic information on the patients in this study.

	Total (n = 364)	Group 1 (n = 295)	Group 2 (n = 69)	P-value
**Age (year)**	29 (27–32)	29 (27–32)	29 (27–32)	0.73
**Height (cm)**	159.0 (155.0–162.0)	160.0 (155.0–162.0)	158.0 (1555.0–162.0)	0.44
**Weight (kg)**	57.0 (52.0–63.0)	57.0 (52.0–63.0)	56.5 (53.5–62.25)	0.58
**BMI (kg/m^2^)**	22.82 (20.58–24.94)	22.46 (20.50–24.97)	22.89 (20.83–24.30)	0.48
**Duration of infertility (year)**	3 (2–5)	3 (2–5)	3 (2–5)	0.96
**Type of infertility [n(%)]**				
Primary	247 (67.9)	198 (67.1)	49 (71.0)	0.53
Secondary	117 (32.1)	97 (32.9)	20 (29.0)
**Irregular menstruation [n(%)]**				
Yes	322 (88.5)	266 (90.2)	56 (81.2)	0.035
No	42 (11.5)	29 (9.8)	13 (18.8)	
**Serum levels of T (ng/mL)**	0.40 (0.31–0.55)	0.41 (0.32–0.55)	0.37 (0.28–0.53)	0.15
**Serum levels of AMH (ng/mL)**	9.75 (5.40–14.76)	10.42 (6.24–14.52)	6.03 (3.61–10.20)	< 0.001
**Serum levels on days 2–3 of the menstrual cycle**				
E2 (pg/mL)	39.4 (30.3–51.0)	39.5 (30.7–48.6)	39.2 (26.1–57.5)	0.90
P (ng/mL)	0.44 (0.31–0.58)	0.44 (0.32–0.55)	0.47 (0.27–0.59)	1.00
LH (IU/L)	7.5 (4.9–12.1)	7.9 (5.0–11.8)	6.6 (3.7–12.9)	0.19
FSH (IU/L)	6.4 (5.5–7.7)	6.4 (5.6–7.7)	6.3 (5.0–7.9)	0.37
LH/FSH	1.26 (0.79–1.88)	1.24 (0.81–1.88)	1.30 (0.58–1.88)	0.47
**PCOM [n(%)]**	233 (64.0)	201 (68.1)	32 (46.4)	0.001
**Fertilization type [n(%)]**				
IVF	311 (85.4)	247 (83.7)	64 (92.8)	0.12
ICSI	12 (3.3)	10 (3.4)	2 (2.9)	
IVF+ICSI	41 (11.3)	38 (12.9)	3 (4.3)	

T, free androgen; AMH, anti-Mullerian hormone; E2, estrogen; P, progesterone; LH, luteinizing hormone; FSH, follicle-stimulating hormone; PCOM, polycystic ovarian morphology; IVF, in vitro fertilization; ICSI, intracytoplasmic sperm injection.

### Laboratory and Clinical Outcomes Between the Two Groups

The clinical outcomes for the PCOS patients in group 1 and group 2 are shown in **Table 2**. The durations of Gn stimulation were shorter in group 1 than in group 2 [10 (9–11) vs. 12 (10–13), p < 0.001], and the total Gn doses in group 1 were also lower [1700.0 (1375.0–2175.0) vs. 2175.0 (1725.0–2800.0), p < 0.001]. On the trigger day, serum E2, P, and LH levels were higher in group 1 than in group 2 [p = 0.001, 0.014 and p < 0.001], but there was no statistical difference in single ET and the number of follicles greater than or equal to 14 mm in diameter. The numbers of oocytes retrieved were similar in both groups [14 (10–20) vs. 13 (10–20), p = 0.71], and there was no statistical difference in the ICSI fertilization rate, cleaved oocyte rate, blastocyst formation rate, available blastocyst rate, high-quality blastocyst rate, or embryo transfer cancellation rate among the two groups. The IVF fertilization and severe OHSS rates were, however, higher in group 2 than in group 1 [82.7% vs. 77.3% and 11.6% vs. 3.7%; p < 0.001 and p = 0.008]. The available D3 embryo rate and the high-quality D3 embryo rate were higher in group 1 than in group 2 [71.4% vs. 66.4% and 50.3% vs. 45.0%; p = 0.004 and 0.012]. Also, although the biochemical pregnancy rate and clinical pregnancy rate showed no statistical differences among the two groups (p = 0.23 and 0.41), these measures still demonstrated a lower trend in group 1 than in group 2 (60.3% vs. 71.9% and 54.3% vs. 62.5%).

**Table 2:** Comparison of clinical outcomes between the two groups.

	Total (n = 364)	Group 1 (n = 295)	Group 2 (n = 69)	P-value
**Duration of Gn (days)**	10 (9–12)	10 (9–11)	12 (10–13)	< 0.001
**Total Gn dose (IU)**	1,800.0 (1,425.0–2,250.0)	1,700.0 (1,375.0–2,175.0)	2,175.0 (1,725.0–2,800.0)	< 0.001
**On the trigger day**				
E2 (pg/mL)	4,134.3 (2,749.0–6,424.0)	4,552.4 (2,813.0–6,652.0)	2,972.1 (2,400.8–4,865.2)	0.001
P (ng/mL)	0.92 (0.65–1.31)	0.96 (0.68–1.33)	0.75 (0.57–1.19)	0.014
LH (IU/L)	1.6 (0.7–3.0)	2.0 (1.0–3.4)	0.5 (0.3–0.7)	< 0.001
Single ET (mm)	5.0 (4.5–5.9)	5.0 (4.5–5.8)	5.5 (4.5–6.0)	0.23
No. of follicles ≥ 14 mm	9 (8–12)	9 (8–13)	10 (8–11)	0.84
**No. of oocytes retrieved**	14 (10–20)	14 (10–20)	13 (10–20)	0.71
**IVF fertilization rate [n(%)]**	4,116/5,252 (78.4)	3,276/4,236 (77.3)	840/1,016 (82.7)	< 0.001
**ICSI fertilization rate [n(%)]**	424/473 (89.6)	392/434 (90.3)	32/39 (82.1)	0.10
**Cleaved oocyte rate [n(%)]**	4,474/4,540 (98.5)	3,612/3,668 (98.5)	862/872 (98.9)	0.40
**Available D3 embryo rate [n(%)]**	3,150/4,474 (70.4)	2,578/3,612 (71.4)	572/862 (66.4)	0.004
**High-quality D3 embryo rate [n(%)]**	1,673/3,395 (49.3)	1,370/2,721 (50.3)	303/674 (45.0)	0.012
**Blastocyst formation rate [n(%)]**	1,605/2,259 (71.0)	1,349/1,890 (71.4)	256/369 (69.4)	0.44
**Available blastocyst rate [n(%)]**	1,396/1,605 (87.0)	1,176/1,349 (97.2)	220/256 (85.9)	0.59
**High-quality blastocyst rate [n(%)]**	466/1,605 (29.0)	387/1,349 (29.7)	79/256 (30.9)	0.48
**Embryo transfer cancelled [n(%)]**	216 (59.3)	179 (60.7)	37 (53.6)	0.28
**Severe OHSS rate [n(%)]**	19 (5.2)	11 (3.7)	8 (11.6)	0.008
**Biochemical pregnancy rate [n(%)]**	93/148 (62.8)	70/116 (60.3)	23/32 (71.9)	0.23
**Clinical pregnancy rate [n(%)]**	83/148 (56.1)	63/116 (54.3)	20/32 (62.5)	0.41

Gn, gonadotropin; E2, estrogen; P, progesterone; LH, luteinizing hormone; ET, endometrial thickness; IVF, in vitro fertilization; ICSI, intracytoplasmic sperm injection; OHSS, ovarian hyperstimulation syndrome.

### A Subgroup Analysis of the Severe OHSS Rate

The results of a subgroup analysis for the severe OHSS rate are shown in **Table 3**. A subgroup analysis based on bLH/FSH shows that, in patients with bLH/FSH levels of at least 1.33, the severe OHSS rate was different between the two groups [OR (95% CI): 5.08 (1.64–15.76), p = 0.005] but was similar in patients with bLH/FSH levels less than 1.33 [OR (95% CI): 1.09 (0.11–10.05), p = 0.94]. The study population was further divided into two subgroups by serum AMH levels; in the AMH > 3.4-ng/ml subgroup, the severe OHSS rate was higher in group 2 [OR (95% CI): 4.42 (1.66–11.76), p = 0.003], but in the AMH ≤ 3.4-ng/ml subgroup, the corresponding statistics could not be calculated because of zero events.

**Table 3:** A subgroup analysis of the severe OHSS rate.

	Group 1 (n = 295)		Group 2 (n = 69)		OR (95% CI)	P-value
	Total	Events (%)	Total	Events (%)		
**bLH/FSH**						
<1.33	161	4 (2.5)	37	1 (2.7)	1.09 (0.11–10.05)	0.94
≥1.33	134	7 (5.2)	32	7 (21.9)	5.08 (1.64–15.76)	0.005
**AMH**						
≤3.4 ng/ml	20	1 (5.0)	13	0 (0)	–	–
>3.4 ng/ml	275	10 (3.6)	56	8 (14.3)	4.42 (1.66–11.76)	0.003

LH, luteinizing hormone; FSH, follicle-stimulating hormone; AMH, anti-Mullerian hormone; OR, odds ratio; CI, confidence interval.

Group 1 = “0”; group 2 = “1.”

### A Subgroup Analysis of the Clinical Pregnancy Rate

The results of a subgroup analysis of the clinical pregnancy rate are shown in **Table 4**. In patients with lower bLH/FSH (<1.33) and lower AMH (≤3.4 ng/ml) levels, group 2 had a higher clinical pregnancy rate compared to group 1 (61.1% vs. 51.3 and 77.8% vs. 64.3%), but this difference did not reach statistical significance [OR (95% CI): 0.67 (0.24–1.92) and 0.51 (0.08–3.49); p = 0.45 and 0.49]. In patients with higher bLH/FSH (≥1.33) and higher AMH (>3.4 ng/ml) levels, the clinical pregnancy rate was similar in both groups [64.3% vs. 60.0% and 56.5% vs. 52.9%, OR (95% CI): 0.83 (0.24–2.95) and 0.87 (0.35–2.15); p = 0.78 and 0.76].

**Table 4:** A subgroup analysis of the clinical pregnancy rate.

	Group 1 (n = 116)		Group 2 (n = 32)		OR (95% CI)	P-value
	Total	Events (%)	Total	Events (%)		
**bLH/FSH**						
<1.33	76	39 (51.3)	18	11 (61.1)	0.67 (0.24–1.92)	0.45
≥1.33	40	24 (60.0)	14	9 (64.3)	0.83 (0.24–2.95)	0.78
**AMH**						
≤3.4 ng/ml	14	9 (64.3)	9	7 (77.8)	0.51 (0.08–3.49)	0.49
>3.4 ng/ml	102	54 (52.9)	23	13 (56.5)	0.87 (0.35–2.15)	0.76

LH, luteinizing hormone; FSH, follicle-stimulating hormone; AMH, anti-Mullerian hormone; OR, odds ratio; CI, confidence interval.

Group 1 = “0”; group 2 = “1.”

## Discussion

As the most common endocrinal disorder characterized by oligo-anovulation, hyperandrogenemia, and polycystic ovaries on ultrasonography, PCOS seriously affects female reproductive health. In addition to reproductive disorders, women with PCOS are also at high risk for other long-term health problems, metabolic complications, and psychological problems, such as type II diabetes mellitus, cardiovascular disease, and anxiety (2). Hence, the diagnosis, prediction, treatment, and prognosis of PCOS deserve the attention of clinicians. For women suffering from PCOS with oligo-anovulation, carefully conducted and monitored pharmacological ovulation induction can be considered. Clomiphene citrate (CC) and letrozole are used as first-line pharmacotherapy, and gonadotropins and laparoscopic surgery appear to be a good alternative as a second-line treatment (13). As PCOS increasingly causes infertility (about 80% of anovulation infertility is caused by PCOS (3)), ART has been widely used in PCOS to help patients achieve pregnancy as a third-line treatment.

COS is an important step in IVF/ICSI, embryo transfer cycles, and, in particular, fresh embryo transfer. The fixed GnRH antagonist protocol and follicular phase GnRH agonist protocol are two important and classical COS protocols with different advantages and disadvantages. A rare but severe complication associated with COS is severe OHSS, which is regarded as related to the overreaction of the ovaries to Gn. Elevated serum AMH levels, multi-follicular development, and a high number of oocytes retrieved are acknowledged risk factors of severe OHSS (9). Higher bLH/FSH has also recently been shown to be associated with severe OHSS (4, 20). PCOS has been regarded as a risk factor of severe OHSS because some clinical features of PCOS typically reflect high ovarian sensitivity, such as high AMH levels and high bLH/FSH levels. The GnRH antagonist protocol can reduce the incidence of severe OHSS compared to the follicular phase GnRH agonist protocol; thus, it has been regarded as a first-line COS protocol for PCOS patients (13). It must be recognized, however, that the GnRH antagonist protocol results in lower clinical pregnancy and lower live birth rates than the GnRH agonist protocol in the general population (12, 14). Compared to the GnRH antagonist protocol, the follicular phase GnRH protocol may achieve better clinical outcomes, which can be explained by its positive effect on endometrial receptivity (22). It can be concluded that the follicular phase GnRH protocol may be an option for PCOS patients with lower AMH levels and lower bLH/FSH levels, especially for patients with a history of poor clinical outcomes.

In this study, we found that the severe OHSS rate in the follicular phase GnRH agonist group was significantly higher than that of the fixed GnRH antagonist group, but no statistical differences were observed in the biochemical pregnancy rates and the clinical pregnancy rates of the two groups. These results are generally consistent with those of previous studies (10, 11, 23). In this study, however, PCOS patients were innovatively stratified into subgroups according to their bLH/FSH and serum AMH levels. It was found that in PCOS women with higher bLH/FSH and higher serum AMH levels, severe OHSS incidence was higher in the follicular phase GnRH agonist group, but among PCOS women with lower bLH/FSH and lower AMH levels, severe OHSS incidence between the two groups was similar. These results indicate that bLH/FSH and serum AMH levels are worth considering when selecting COS protocols for PCOS patients. Regarding subgroup analyses of clinical pregnancy rates, it seems that no statistically valuable indicator has shown to be a good reference for COS selection in PCOS. Combining the results of these two subgroup analyses, the wild guess that follicular GnRH agonist protocols may be considered as an alternative choice for PCOS patients with lower bLH/FSH and lower serum AMH levels was entertained in this study, as such protocols do not increase the risk of severe OHSS. This assumption must be based, however, on the close observation of OHSS risk, and other OHSS risk factors must be fully considered. The cutoff values of bLH/FSH and serum AMH levels that predict severe OHSS should be verified with large-sample studies, and highly sensitive cutoff values should be selected.

We attempted to explain the results of this study by reviewing related physiological mechanisms. LH and FSH are both pituitary gonadotropin hormones essential for female fertility, and they are regulated by the frequency of pulsatile GnRH. According to the two-cell theory, LH stimulates follicular theca cells to produce androstenedione, and FSH stimulates the synthesis of aromatase in granulosa cells, thus catalyzing the conversion of androstenedione to estradiol. LH and FSH work together to stimulate sex hormone secretion and oocyte development in the ovaries. On days 2–3 of the menstrual cycle, the dominant follicle continues to mature in physiological status under FSH. Increased LH levels can trigger ovarian follicular theca cells to secrete more androgen during this period, and FSH can trigger granulosa cells to convert extra androgens to estrogen (24). Thus, high bLH/FSH has been shown to impair the formation of follicles (25). In PCOS patients receiving GnRH antagonist protocols, endogenous LH was not suppressed during the early stages of Gn stimulation; thus, potential ovarian overstimulation may be inhibited by endogenous LH. In PCOS patients receiving follicular phase GnRH agonist protocols, however, endogenous LH generation is inhibited, and the body loses the potential mechanism of inhibiting ovarian overstimulation due to the downregulated pituitary function. AMH is produced in granulosa cells by pre-antral and small antral follicles and is highly correlated with bLH/FSH in PCOS women (26, 27). For this reason, AMH is also considered to represent ovarian reactivity. For PCOS patients with higher bLH/FSH levels or higher serum AMH levels, the ovaries are more like to respond to Gn, and the GnRH antagonist can thus restrict this reactivity *via* endogenous LH. However, for PCOS women with lower bLH/FSH levels and lower serum levels of AMH, the reactivity of their ovaries to Gn is not as obvious. These mechanisms may explain why the incidence of early-stage OHSS does not show significant differences among PCOS patients with lower bLH/FSH levels and lower AMH levels between the two COS protocols. Also, late-stage OHSS is strongly associated with pregnancy and is restricted to cycles in which clinical pregnancy occurred. PCOS is a strong risk factor of late-stage OHSS because the risk factor for high bLH/FSH levels and high AMH levels among PCOS patients with lower bLH/FSH and lower AMH levels is offset to some extent. Therefore, for PCOS patients with lower bLH/FSH levels and lower serum AMH levels, the follicular phase GnRH agonist protocol may be a viable choice.

It is worth emphasizing that there are some limitations that restrict the credibility of this study. Its results must therefore be carefully interpreted. The most important limitation is its small sample size, which is especially true for its PCOS patients who received the follicular phase GnRH agonist protocol. Because of guidelines published in recent years (13), GnRH antagonist protocols have been widely used in PCOS patients even though the individual differences of PCOS are not discussed in these guidelines. This could explain why there were significantly fewer patients in this study’s follicular phase GnRH agonist group as compared to its GnRH antagonist group. In addition, severe OHSS is a rare complication of COS, and severe OHSS events in some subgroups are numbered as low as zero. All these factors restricted the sample size of this study, but considering the rarity of the resulting events and the interpretability of the results, these results are still worth reporting. Another limitation is due to the inherent nature of retrospective studies, as some potential confounding factors were not excluded in this study. In addition, other clinical outcomes such as the miscarriage rate and the live birth rate were not acquired. In the opinion of this study’s authors, the greatest value of this study is that it provides another choice for controlled ovarian stimulation for PCOS patients with lower bLH/FSH and lower serum AMH levels. The results of this study must be validated by prospective studies with larger samples in the future.

In conclusion, GnRH antagonist protocols should serve as first-line COS protocols for PCOS patients undergoing IVF/ICSI and fresh embryo transfer cycles, but the limited evidence of this study suggests that for PCOS patients with lower bLH/FSH (<1.33) and lower serum AMH levels (≤3.4 ng/ml), follicular phase GnRH agonist protocols may be another safe choice that does not increase severe OHSS risks. The results of this study must be interpreted with caution.

## Data Availability Statement

The raw data supporting the conclusions of this article will be made available by the authors, without undue reservation.

## Ethics Statement

The studies involving human participants were reviewed and approved by the Ethics Committee of West China Second University Hospital. The patients/participants provided their written informed consent to participate in this study.

## Author Contributions

All authors contributed to this study’s conception and design. Material preparation, data collection, and analysis were performed by RG, XL, WH, RZ, and LQ. The first draft of the manuscript was written by RG and PB, and all authors commented on previous versions of the manuscript. All authors contributed to the article and approved the submitted version.

## Conflict of Interest

The authors declare that this research was conducted in the absence of any commercial or financial relationships that could be construed as a potential conflict of interest.

## Publisher’s Note

All claims expressed in this article are solely those of the authors and do not necessarily represent those of their affiliated organizations, or those of the publisher, the editors and the reviewers. Any product that may be evaluated in this article, or claim that may be made by its manufacturer, is not guaranteed or endorsed by the publisher.
